# Not all 5′-deoxyadenosines are created equal: Tracing the provenance of 5′-deoxyadenosine formed by the radical *S*-adenosyl-L-methionine enzyme 7-carboxy-7-deazaguanine synthase

**DOI:** 10.1016/j.jbc.2025.108347

**Published:** 2025-02-25

**Authors:** Saswata Nayak, Andrew S. Jochimsen, Vahe Bandarian

**Affiliations:** Department of Chemistry, University of Utah, Salt Lake City, Utah, USA

**Keywords:** radical SAM enzymes, enzyme mechanism, catalysis, radical intermediate, mechanistic enzymology

## Abstract

Members of the radical *S*-adenosyl-L-methionine (rSAM) enzyme superfamily cleave SAM to generate the highly reactive 5′-deoxyadenosyl radical (dAdo·), where dAdo· initiates the reaction by an H-atom transfer from the substrate to form 5′-deoxyadenosine (dAdo) in nearly every member of the superfamily. However, in all rSAM enzymes, SAM also undergoes reductive cleavage to form dAdo in a reaction uncoupled from the product's formation. Herein, we examine the dAdo that is formed under catalytic conditions with the rSAM enzyme 7-carboxy-7-deazaguanine synthase (QueE), which catalyzes the radical-mediated transformation of 6-carboxy-5,6,7,8-tetrahydropterin (CPH_4_) to 7-carboxy-7-deazaguanine (CDG). We propose that the dAdo that is observed under catalytic conditions can be traced to multiple shunt pathways, which are not all truly uncoupled from catalysis. Indeed, in one case, we demonstrate that the dAdo can form due to the reductive quenching of the initially generated substrate radical by the very same reducing system used to reductively cleave SAM to initiate catalysis. The insights from this work are generally applicable to all members of the rSAM family, as they influence the choice of reducing system to avoid the non-productive shunt pathways that interfere with catalysis.

Radical SAM (rSAM) metalloenzymes comprise a functionally diverse superfamily that is characterized by its use of *S*-adenosyl-L-methionine (SAM) to catalyze challenging radical-mediated transformations (1, 2, 3, 4, 5, 6, 7, 8, 9, 10). In the active site of the rSAM enzymes, three Cys thiolates are present in a highly conserved CxxxCxxC motif (11) that coordinates three iron atoms of a site-differentiated (4Fe-4S) cluster while the fourth iron coordinates SAM through its α-amino and α-carboxylate moieties (12, 13, 14, 15). When reduced from a +2 to the +1 redox state, the RS cluster facilitates the cleavage of the bound SAM cofactor, resulting in the formation of a 5′-deoxyadenosyl radical (dAdo·) and L-methionine (L-Met) (Fig. 1) (16, 17, 18, 19, 20, 21, 22, 23, 24, 25, 26, 27, 28). The dAdo· typically functions as a radical initiator by abstracting an H-atom from the substrate to commence the catalytic cycle.

**Figure 1:**
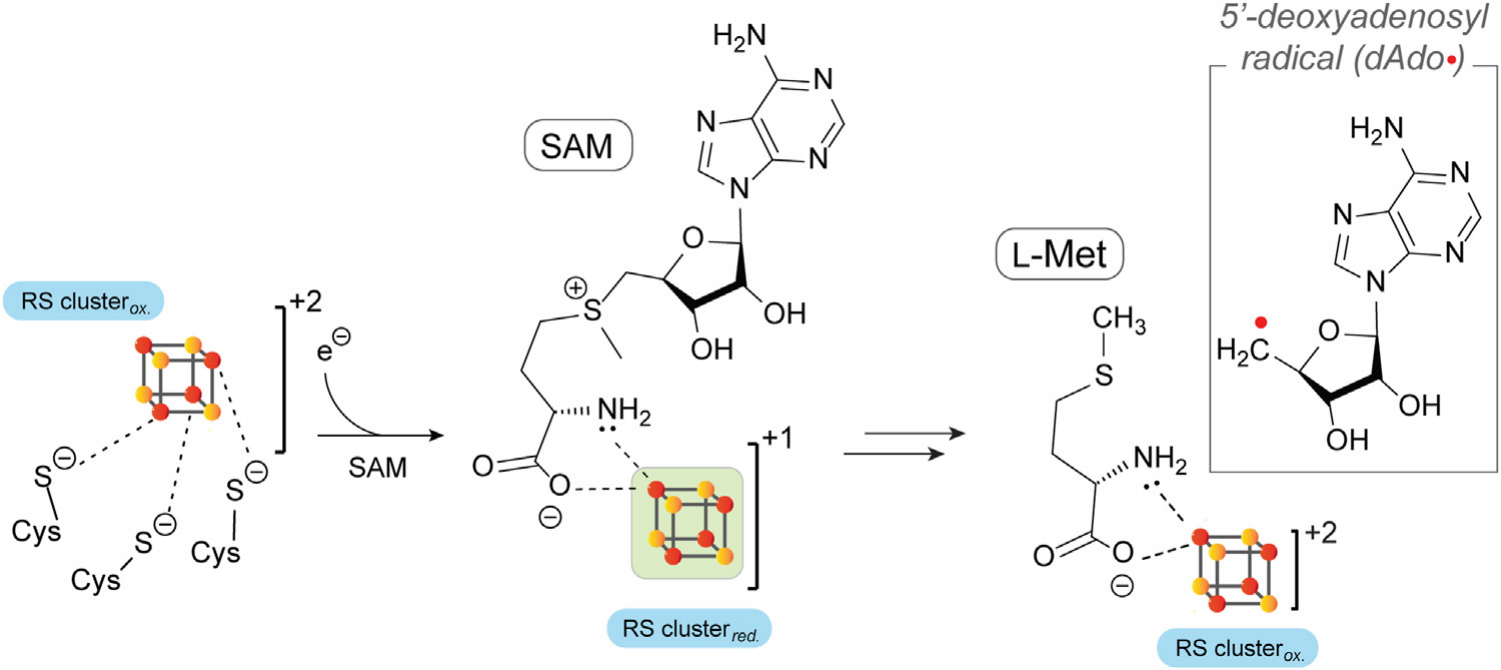
**Reductive cleavage of SAM to generate dAdo·.** The reduction of the [4Fe-4S] cluster from the +2 to the active +1 state (highlighted in *green*) facilitates the cleavage of SAM and the formation of dAdo·.

The reduction in the RS cluster to the +1 state from its inactive +2 state is an essential step in the catalytic cycles of all rSAM enzymes. However, the identity of the *in vivo* reducing partner responsible for this reductive activation of SAM remains elusive. *In vitro* studies typically employ inorganic reductants like sodium dithionite (DT) or titanium (III) citrate, or a system consisting of flavodoxin/flavodoxin reductase with reducing equivalents derived from nicotinamide adenine dinucleotide phosphate (NADPH) (29, 30, 31, 32, 33, 34, 35, 36, 37, 38, 39). This widely used flavodoxin/flavodoxin reductase system is often referred to as the “biological” reducing system (35). However, a recent study suggests that any redox partner capable of shuttling a single electron can adequately fulfill the role of a reducing system (40). Indeed, the “biological” reducing system can be substituted with flavin mononucleotide (FMN) and NADPH (40, 41, 42). Furthermore, it has been proposed that *in vivo,* the redox partner could be any redox-active protein capable of one electron reduction, including other rSAM enzymes. Consistent with this, the same study showed that two unrelated rSAM enzymes could activate each other (40).

While the formation of dAdo· is essential to initiate the catalytic cycle, it is not always coupled with turnover. Briefly, in the absence of substrate and under reducing conditions, rSAM enzymes cleave SAM to form 5′-deoxyadenosine (dAdo) in a process that has been referred to as *abortive cleavage* and is uncoupled from the formation of product (43, 44, 45, 46, 47). Additionally, multiple studies have shown that in certain rSAM enzymatic reactions, the product is not formed when DT is used as the reducing agent, even though dAdo formation can be observed (32, 34). In some cases, substituting DT with the “biological” reducing system, titanium (III) citrate, or the introduction of a redox mediator like methyl viologen in tandem with an electron donor like DT or NADPH was subsequently shown to be more effective for the formation of *both* dAdo *and* product (33, 34, 42, 48, 49, 50). These observations are difficult to explain. While a similar trend is also observed with the B_12_-dependent rSAM enzymes, the distinction with other rSAM enzymes is that DT interferes with the B_12_ cofactor and is incompatible as a reducing agent to study these systems (37, 51, 52, 53, 54). The fact that the inclusion of DT in the rSAM enzyme reactions can lead to formation of dAdo but not product suggests that DT might play an additional role that potentially suppresses (or masks) the catalytic process. Studies reporting this uncoupled dAdo production noted that in reactions carried out with D_2_O, the resulting dAdo can incorporate a solvent-derived deuterium, suggesting that an H-atom transfer reaction that quenches dAdo· is taking place *via* a solvent exchangeable site (34, 48, 55).

The dAdo that forms in the presence of substrate, even when the product is not observed, can arise from two scenarios. In the first, the initially formed dAdo· is rapidly quenched to form dAdo if there is no substrate productively bound in the active site. Alternatively, the initially formed substrate radical could be intercepted and quenched by an exogenous reductant, leading to the premature release of dAdo. This is consistent with the observation that the inclusion of more selective or hindered reductants, such as flavodoxin/flavodoxin reductase/NADPH, leads to product formation (32, 48). To date, there have not been efforts to systematically differentiate between dAdo formed in abortive cleavage *versus* as a shunt product resulting from the capture of a radical intermediate.

The rSAM enzyme QueE catalyzes a crucial radical-mediated ring contraction step in the biosynthesis of 7-deazapurine-containing natural products. The catalytic cycle is initiated with an H-atom transfer from the C-6 position of 6-carboxy-5,6,7,8-tetrahydropterin (CPH_4_) to dAdo·, followed by radical-mediated rearrangements that produce 7-carboxy-7-deazaguanine (CDG) and regenerate SAM (Fig. 2) (31, 33, 55, 56, 57). A *gem*-aminocarboxylate intermediate is proposed to be the initial product of the reaction, which then undergoes stereoselective deprotonation and elimination of ammonia to form the final product CDG. Like other rSAM enzymes, QueE also catalyzes abortive cleavage of SAM, as evidenced by the formation of a pool of unlabeled dAdo when C-6-deuterated CPH_4_ ([6-D] CPH_4_) is used as the substrate (55). Conversely, when the reaction is carried out with unlabeled CPH_4_ in the presence of D_2_O in the same study, the resulting dAdo consists mainly of *unlabeled* and *singly* deuterated species. Because QueE utilizes SAM catalytically and forms dAdo in both the presence or absence of a substrate, it can serve as a model system to understand the enigmatic observations about the relationship between the reductant and the formation of dAdo.

**Figure 2:**
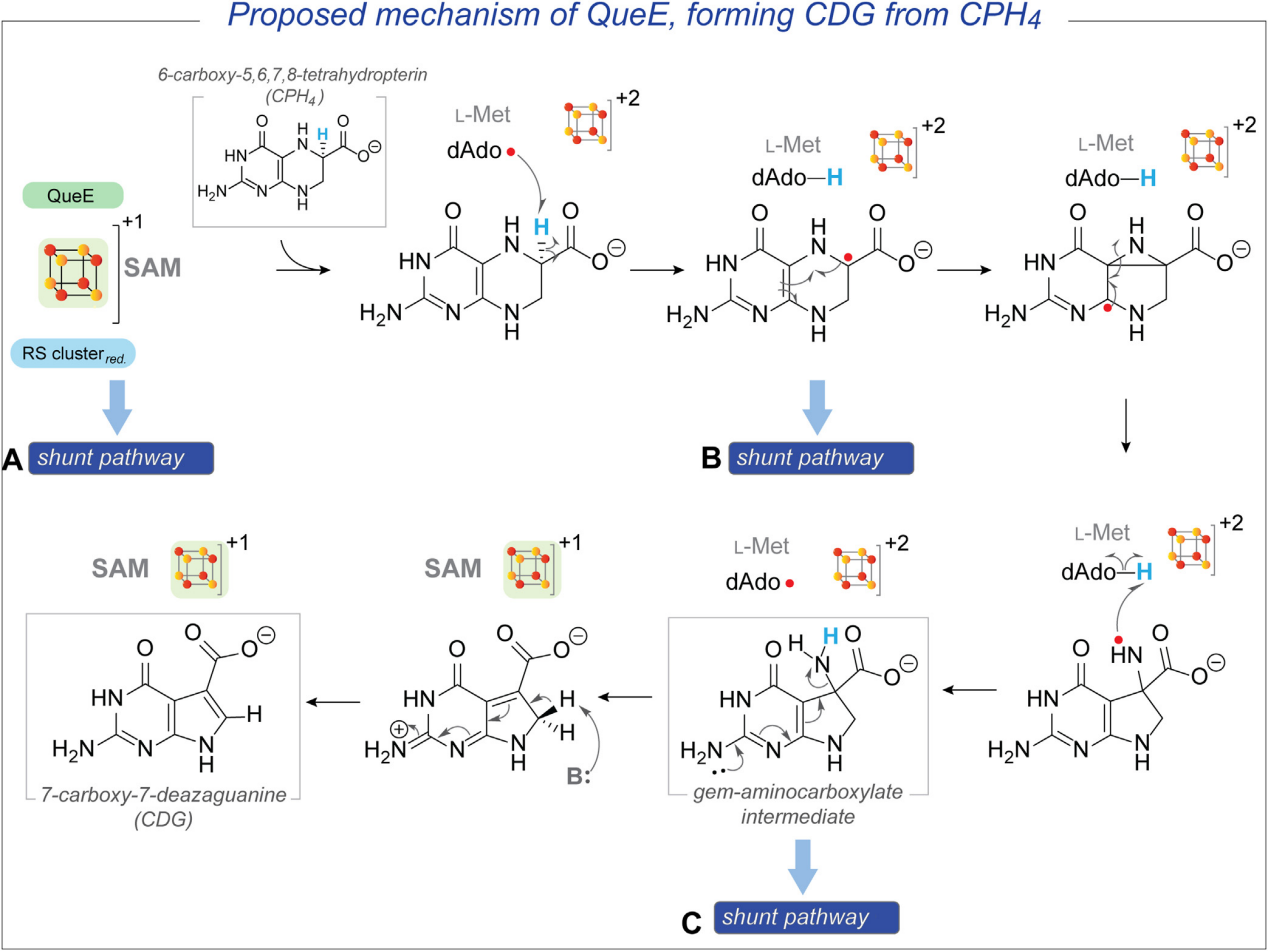
**QueE catalyzes a radical-mediated ring contraction of CPH_4_ to form CDG.** This figure shows the proposed *gem*-aminocarboxylate intermediate in the mechanism of forming CDG from CPH_4_. The possible shunt pathways that can produce dAdo are highlighted (A) the dAdo· generated by the reductive cleavage of SAM can be quenched in the absence of the CPH_4_ substrate *via* the abortive cleavage pathway, generating dAdo in the process, (B) the substrate radical can be captured by reduction present in the reaction, which can then get quenched, and (C) the premature reduction of the RS cluster can lead to the intermediate to stall in the active site, leading to exchange of protons between dAdo· and the amine group of the *gem*-aminocarboxylate intermediate.

Herein, we report the deconvolution of the source of dAdo that is formed by QueE in the presence and absence of substrate by building on the serendipitous observation that the dAdo formed under certain reducing conditions in reaction mixtures containing D_2_O is *multiply* deuterated. Follow-up experiments show that dAdo formed in the reaction of QueE likely originates from one of at least three pathways (highlighted in Fig. 2). One pathway involves the abortive cleavage of SAM (Fig. 2, shunt pathway **A**) while another involves the reductive capture of the initially formed substrate radical (Fig. 2, shunt pathway **B**). Alternatively, in QueE, reforming SAM after each catalytic cycle requires an H-atom transfer from the dAdo generated during the reaction to form the proposed *gem*-aminocarboxylate intermediate (see Fig. 2). Premature reduction of the RS cluster could inhibit the regeneration of SAM formation, leading to dAdo accumulation (Fig. 2, shunt pathway **C**). The implications of these results for catalysis and activation are discussed.

## Results

### Multiply deuterated dAdo forms during turnover in D_2_O

**Figure 3:**
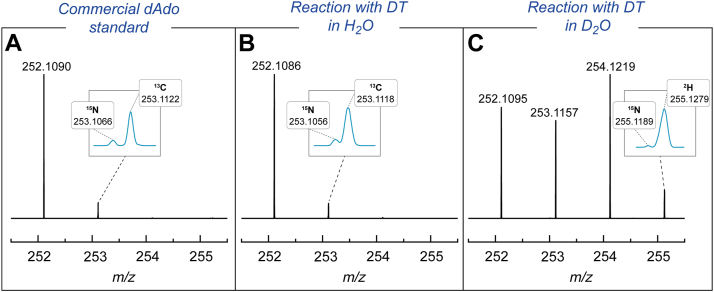
**Multiply deuterated dAdo is observed in the reaction with QueE and CPH_4_ in D_2_O, with DT as the reductant.** Mass spectral data of (*A*) commercial dAdo standard compared to (*B*) dAdo obtained when QueE reaction with CPH_4_ is carried out in H_2_O or (*C*) in >95% D_2_O.

Previous studies have demonstrated that rSAM enzymes, including QueE, can form deuterated dAdo when the reaction is performed in D_2_O (48, 55). To investigate this phenomenon, QueE was incubated with SAM under catalytic conditions. In these initial assays, 2 mM DT was used as the source of the reducing equivalent to activate QueE. The assays contained 50 mM potassium phosphate (KPi) buffer pH or pD 7.4, 2 mM MgSO_4_, 2 mM CPH_4_, 25 μM QueE, and 2 mM SAM in D_2_O, with control reactions performed in H_2_O. Each reaction mixture was incubated for 18 h, quenched with 3% trichloroacetic acid (TCA) (*w/v*), and analyzed by UHPLC-MS. Earlier studies with QueE had utilized assay conditions with 60% D_2_O (55). In the experiments reported here, the D_2_O content in the reaction was generally maintained at >95% unless stated otherwise. The reaction mixtures were analyzed using an LC-MS method capable of separating SAM, dAdo, CPH_4_, and CDG (see Fig. S1). The data indicate that dAdo forms in the incubation, as evidenced by a peak in the chromatogram (Fig. S2) with a retention time similar to that of authentic dAdo standard that exhibits an absorbance maximum at 258 nm and a monoisotopic mass at *m/z* 252.1086 (Fig. 3*A*, C_10_H_13_N_5_O_3_, theoretical [M+H]^+^ *m/z* 252.1091). In the MS data of the reactions carried out in H_2_O, the natural abundance ^15^N and ^13^C isotopic peaks of dAdo are also well resolved at *m/z* 253.1056 and 253.1118, respectively, and are all within 3 ppm of the expected values (Fig. 3*B*, ^15^**N**_1_C_10_H_13_N_4_O_3_, theoretical [M+H]^+^ *m/z* 253.1061; ^13^**C**_1_C_9_H_13_N_5_O_3_, theoretical [M+H]^+^ *m/z* 253.1125).

In contrast to the reactions conducted in H_2_O, when QueE is incubated under similar conditions as above but in >95% D_2_O, the dAdo formed is multiply deuterated (see Fig. 3*C* for MS data). In these reactions, in addition to the peak for unlabeled dAdo (C_10_H_13_N_5_O_3_, observed 252.1086, theoretical [M+H]^+^ *m/z* 252.1091), singly deuterated dAdo (^2^**H**_1_C_10_H_12_N_5_O_3_, observed *m/z* 253.1157, theoretical [M+H]^+^ *m/z* 253.1154) and doubly deuterated dAdo (^2^**H**_2_C_10_H_11_N_5_O_3_, observed *m/z* 254.1219, theoretical [M+H]^+^ *m/z* 254.1217) are also present. Additionally, there is also a peak at *m/z* 255.1279, consistent with a triply deuterated dAdo species (see inset of Fig. 3*C*, ^2^**H**_3_C_6_H_10_N_5_O_3_, theoretical [M+H]^+^ *m/z* 255.1279). The observed *m/z* values for the deuterated species are all within 3 ppm of the theoretical values. It should be noted that for all the deuterated dAdo species, the natural abundance ^15^N and ^13^C peaks, though relatively small, are also observed. The ^15^N isotopic peak is generally resolved from the peak of the deuterated species; however, the ^13^C isotopic peak is not. The theoretical contribution of the ^13^C peak is accounted for (see Experimental procedures) before calculating the amount of deuterated dAdo in the observed peak. Multiply deuterated species are observed at all time points of the reaction in D_2_O with DT (Figs. S3 and S4).

The protons on the C-2 and C-8 carbons of the adenosine base in dAdo are known to undergo slow exchange in D_2_O (58, 59, 60). To rule out the possibility that multiply deuterated species arose from proton exchange with the adenine base of dAdo, overnight control reactions containing commercial dAdo, L-Met, QueE, CPH_4_, and DT were prepared in D_2_O. rSAM enzymes are not capable of generating dAdo· from dAdo and L-Met; therefore, any observed deuteration of dAdo would have resulted from exchange with solvent. The LC-MS analysis of these control reactions showed very little deuterium incorporation into dAdo (Fig. S5), thus eliminating the possibility of the deuterium exchange between the base and the solvent as the source of deuterations on dAdo.

**Figure 4:**
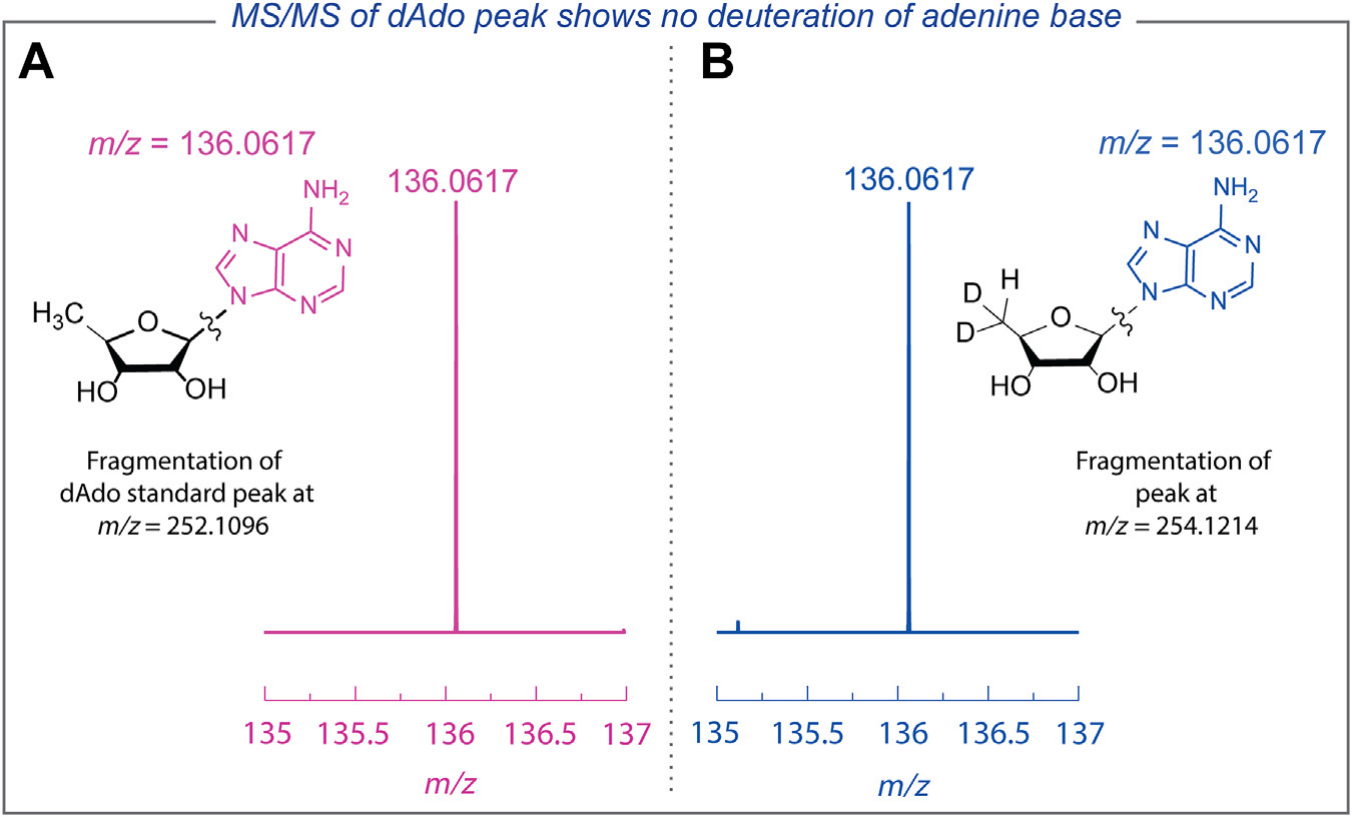
**Localizing the site of deuteration to the ribose moiety of dAdo.** Positive ion mode MS/MS fragmentation spectrum of (A) commercial dAdo standard (*m/z* 252.1096) is compared to (B) doubly deuterated dAdo species (*m/z* 254.1214) from reactions carried out in D_2_O.

To determine the site of deuterium incorporation on dAdo (ribose or base), an MS/MS analysis was carried out with the dAdo generated from catalytic reactions conducted in D_2_O. All species identified in the range of *m/z* 252.1217 to 257.1217 were isolated in the ion trap of the mass spectrometer, and the ion with the desired mass was isolated further and fragmented. Controls with commercial dAdo (*m/z* 252.1096) showed that the fragmentation occurs at the N-glycosidic bond to release a fragment corresponding to the base at *m/z* 136.0617 (Fig. 4*A*). An identical fragment was also observed when the doubly deuterated dAdo species (*m/z* 254.1214) was isolated and fragmented in the mass spectrometer, thus narrowing the site of labeling to the ribose moiety of dAdo, presumably at the C-5′ position. These controls demonstrate that the deuterated dAdo observed when QueE and CPH_4_ are incubated under reducing (catalytic) conditions originates from reductive cleavage of SAM and not background exchange with the adenine base.

### Multiple deuteration of dAdo occurs predominantly with DT as the reducing agent

**Figure 5:**
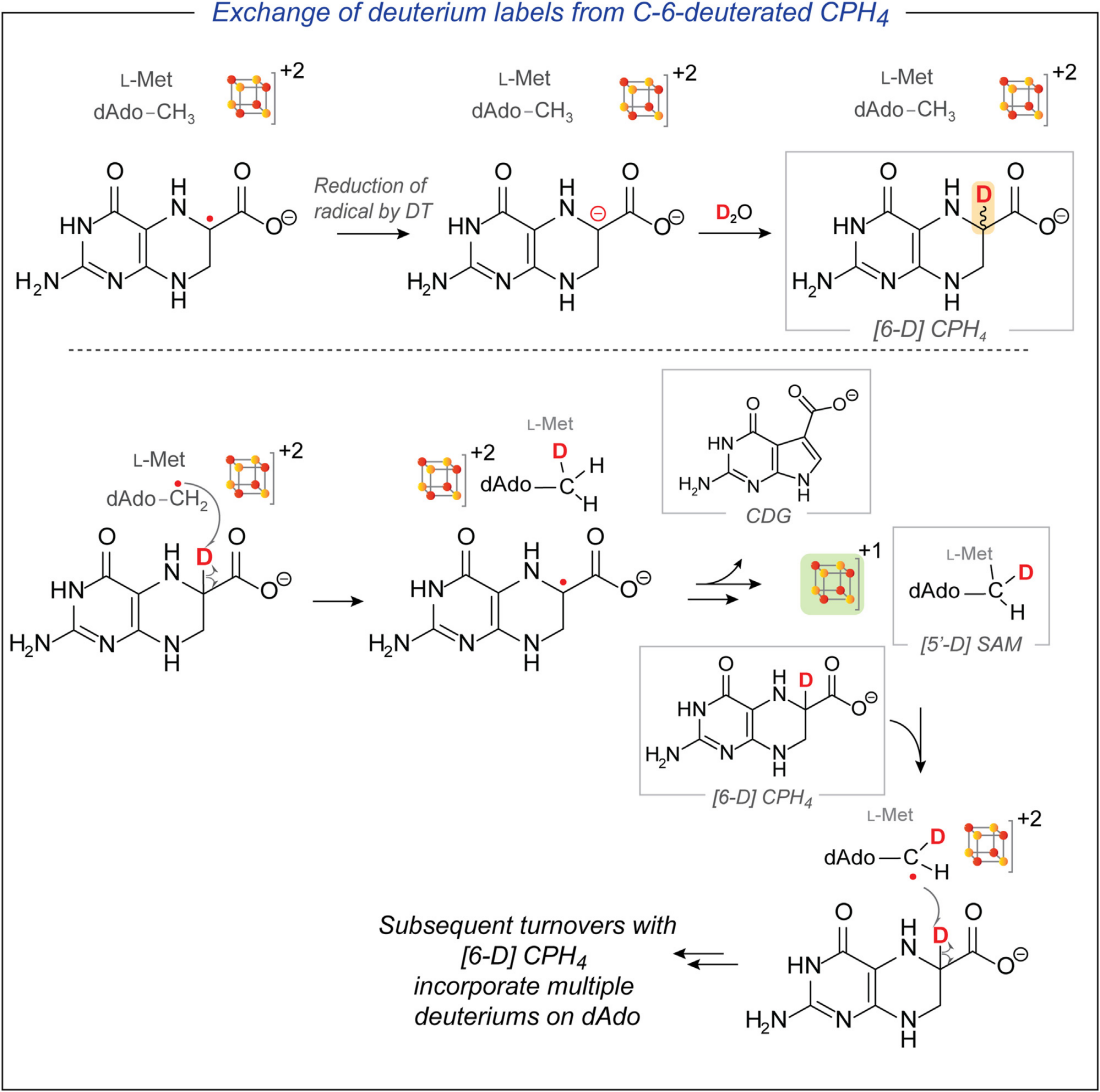
**Reduction of the CPH_4_ radical intermediate can lead to deuterated CPH_4_**. In a subsequent turnover cycle, the deuterium label can be incorporated into dAdo. This paradigm shows how dAdo can become multiply deuterated and bulk CPH_4_ can be enriched in deuterium.

**Figure 6:**
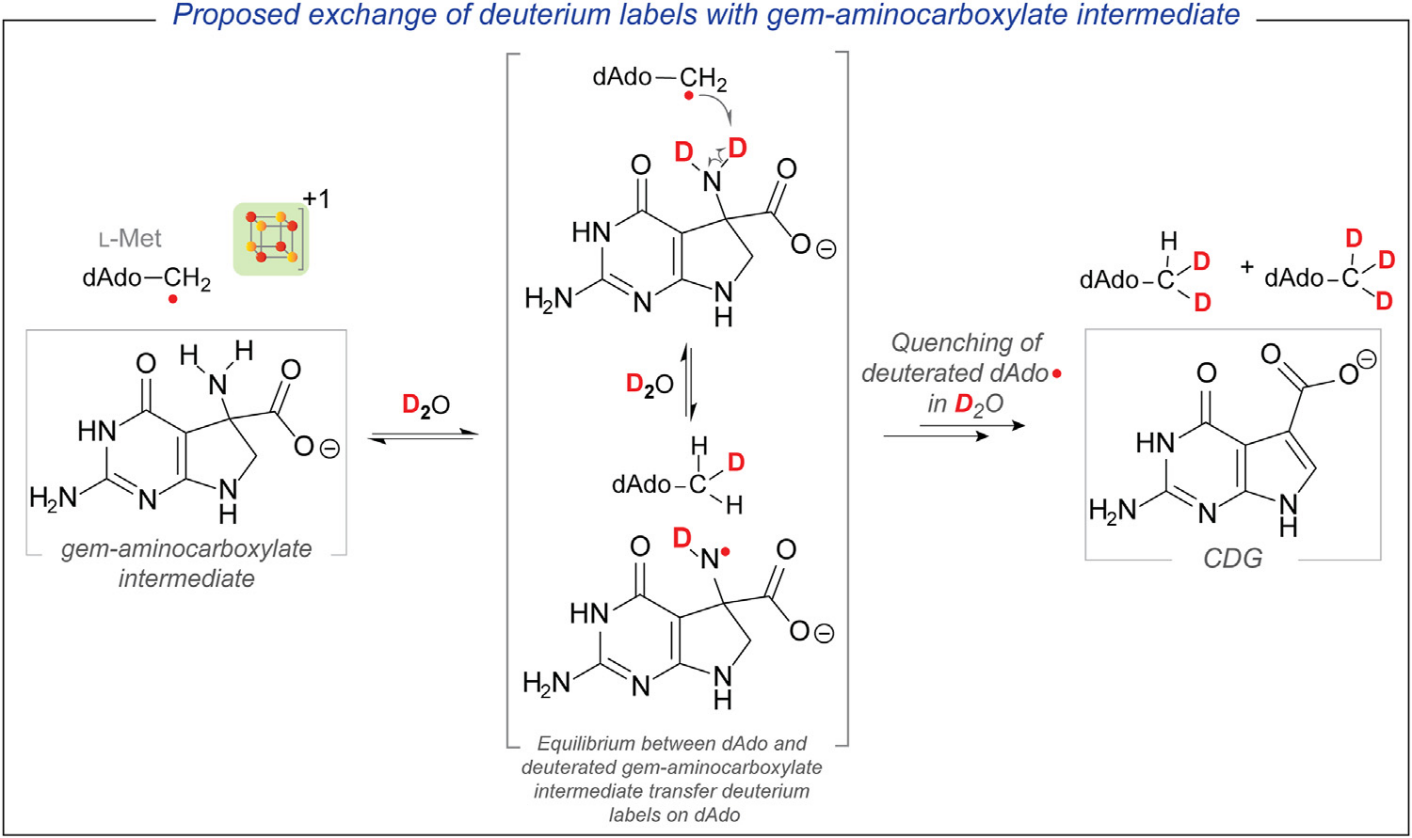
**DT can potentially reduce the RS cluster prematurely.** A reduced cluster cannot catalyze the reformation of SAM in the active site. Under these conditions, the amino group of the proposed *gem*-aminocarboxylate intermediate, which is solvent exchangeable, can exchange deuterium with dAdo·, eventually leading to the formation of multiply deuterated dAdo.

Next, we considered two paradigms that could account for the formation of multiply deuterated dAdo under catalytic conditions (Fig. 5 and 6). QueE catalyzes the ring contraction by initiating H-atom abstraction from the C-6 position of CPH_4_ to generate a substrate radical. In one pathway, the initially formed CPH_4_ C-6 radical intermediate could be intercepted by DT and quenched by solvent-derived deuterium to form [6-D] CPH_4_ (Fig. 5). While this pathway itself would not lead to the deuteration of dAdo directly, it creates the opportunity for [6-D] CPH_4_ to participate in a second round of catalysis. Since QueE uses SAM catalytically, after one turnover with [6-D] CPH_4_, the now-deuterated dAdo· would recombine with L-Met to form C-5′-deuterated SAM ([5′-D] SAM). If this [5′-D] SAM participates in additional turnover cycles with [6-D] CPH_4_, it can incorporate a second deuterium. Additionally, the deuterated dAdo· species generated from this molecule of SAM can potentially be quenched by a solvent-derived proton (or deuterium in D_2_O) to form dAdo containing one, two, or three deuterium labels.

Alternatively, the initially proposed substrate radical can undergo a ring contraction to a product-like radical before abstracting an H-atom from dAdo to form the *gem*-aminocarboxylate intermediate. The amino group of these intermediates may undergo deuterium exchange with solvent-derived deuterons (Fig. 6). Additionally, since QueE uses SAM catalytically, the initially formed dAdo· can combine with L-Met to reform SAM at the end of the catalytic cycle while reducing the oxidized [4Fe-4S]^+2^ rSAM cluster back to the +1 state. However, if this oxidized [4Fe-4S]^+2^ cluster is prematurely reduced by DT instead while the enzyme is engaged in catalysis, the dAdo· would not be able to reform SAM, thus stalling the conversion to product. In this scenario, the dAdo· may undergo reversible H-transfer events with the deuterated amino group of the *gem*-aminocarboxylate intermediate and incorporate multiple deuterium labels.

In both scenarios depicted in Figs. 5 and 6, the underlying assumption is that DT intercepts dAdo·, the CPH_4_ intermediate, or the oxidized cluster. If so, then changing the source of reducing equivalents that are used to activate QueE may suppress the formation of multiply deuterated dAdo. Therefore, experiments were carried out with two alternate reducing systems. In the first, *Bacillus subtilis* flavodoxin/flavodoxin reductase (YkuN/FPR) homologs were used to reductively activate QueE with reducing equivalents derived from NADPH, which served as the “biological” reducing system. In the second, FMN and NADPH were employed as the reductant, which has recently been shown to reduce QueE and a different rSAM enzyme, PapB (40). In these experiments, DT was replaced with either 25 μM YkuN/25 μM FPR/2 mM NADPH or 50 μM FMN/2 mM NADPH. Control experiments with 2 mM DT were also included for comparison. The D_2_O content in these reactions was maintained at 78 to 95%.

**Figure 7:**
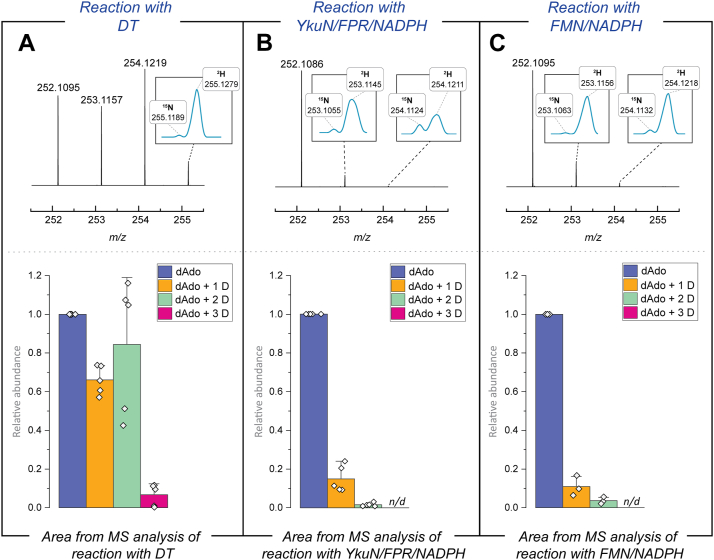
**The formation of multiply deuterated dAdo is linked to the identity of the reducing system.** Assay mixtures contained QueE, SAM, CPH_4,_ and (A) DT, (B) YkuN/FPR/NADPH, or (C) FMN/NADPH to reductively activate QueE. The area under the peaks in the EICs is calculated and averaged over at least three replicates (see Experimental procedures). In each set of experiments, the area under the EIC of the deuterated dAdo species was normalized to that of unlabeled dAdo. The error bars represent standard deviation from at least three replicates.

LC-MS analyses of the dAdo produced during reactions, where DT, YkuN/FPR/NADPH, or FMN/NADPH were used to reductively activate QueE, are shown in Figure 7. The pool of dAdo that forms in the positive control containing DT consists of 40% unlabeled, 24% singly deuterated, 33% doubly deuterated, and 3% triply deuterated dAdo (as calculated from the average EIC area from the MS data analysis in Fig. 7*A*). By contrast, >90% of the dAdo species produced with the alternate reducing systems are unlabeled, with singly deuterated dAdo dominating the labeled pool of dAdo (see Fig. 7, *B* and *C*). The presence of deuterium is unambiguously established in each sample by examining the corresponding MS peak, which is resolved from that of the ^15^N isotope (see inset MS figures in Fig. 7). We note that the peak for the deuterated species is not well resolved from that of natural abundance ^13^C, so the percentages for deuterated material represent upper limits in each case. No triply deuterated species were observed in reactions containing alternate reductants.

CPH_4_ is prone to oxidation when exposed to light and oxygen, with the dihydropterin and pterin forms serving as potential sources of deuterated substrate under reducing conditions in D_2_O. Limited degradation of CPH_4_ is unavoidable during purification, even if all the steps are carried out anaerobically and in the dark. To account for this background, a reaction mixture containing a stock of CPH_4_ with significant quantities of oxidized forms (∼30% oxidized, as characterized by MS) was incubated with DT in D_2_O under the same assay conditions described previously, but without QueE. After 18 h of incubation at room temperature, the assays were quenched with 3% TCA and analyzed on the mass spectrometer. While retention time controls corresponding to the dihydro and the pterin forms were not available, extracted ion chromatograms show evidence for the presence of both in the stock solution (pterin form, theoretical [M+H]^+^ *m/z* = 208.0465; dihydropterin form, theoretical [M+H]^+^ *m/z* = 210.0621; CPH_4_, theoretical [M+H]^+^ *m/z* = 212.0778). The area under the corresponding peaks was monitored over extended incubation times (see Fig. S6). The data show little change in the amounts of the oxidized forms even after 18 h of incubation in the assay mixture, which rules out reduced dihydro and pterin forms as the source of the multiply deuterated dAdo.

**Figure 8:**
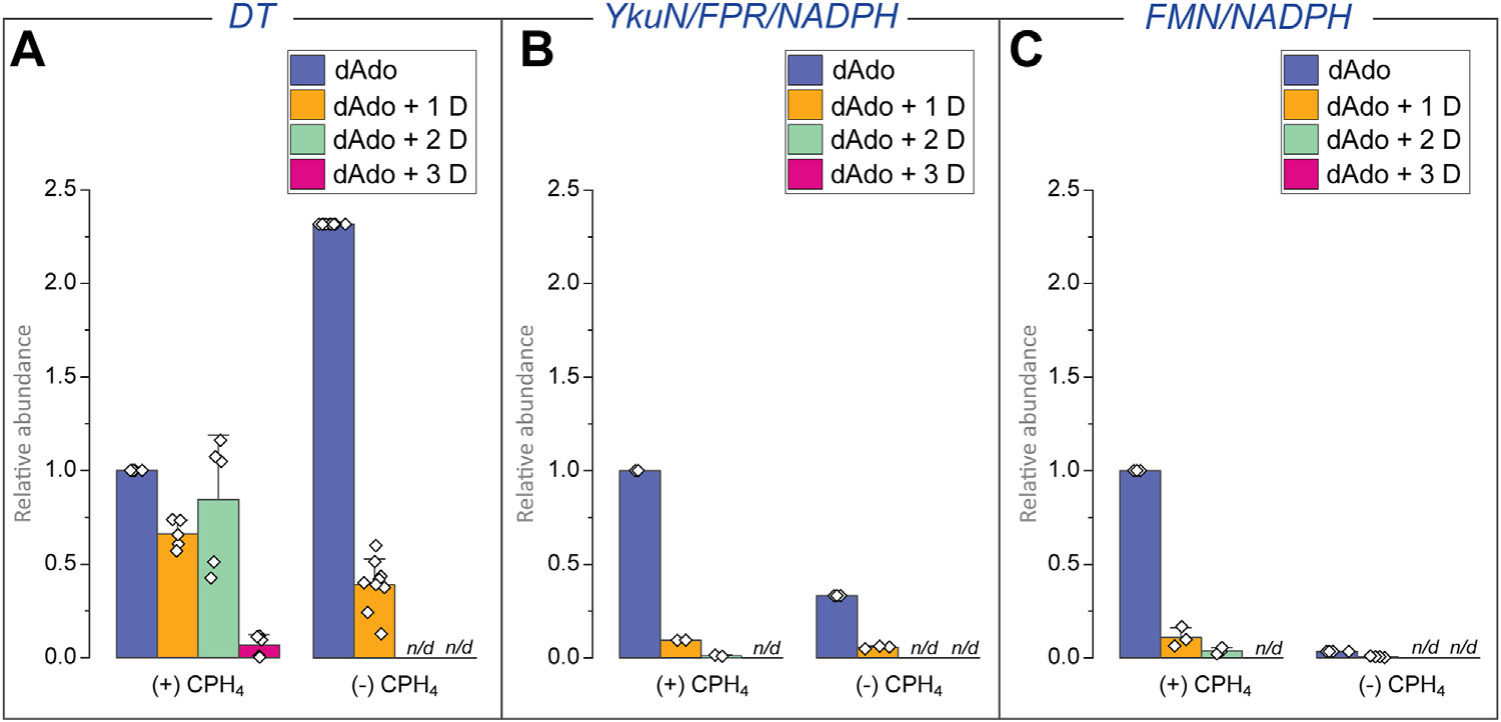
**Comparison of deuterium content of dAdo in the presence or absence of substrate and as a function of reductant identity.** The reactions were carried out in the presence and absence of CPH_4_ with (A) DT, (B) YkuN/FPR/NADPH, or (C) FMN/NADPH to reductively activate QueE. The amounts of dAdo formed in the absence of CPH_4_ are normalized to the dAdo formed in the reaction with CPH_4_ and are averaged over at least three replicates. In each set of experiments, the area under the EIC of the deuterated dAdo species was normalized to that of unlabeled dAdo. The error bars represent standard deviation from at least three replicates. Additionally, the area of the EIC for the control reaction dataset with no CPH_4_ was normalized to the area corresponding to the reaction dataset containing CPH_4_.

In the experiments described to rationalize the multiply deuterated dAdo above, we cannot distinguish the pools of dAdo that are formed uncoupled to catalysis *via* either the abortive cleavage pathway or other shunt pathways shown in Figs. 5 and 6. The deuteration of dAdo produced in the presence or absence of CPH_4_ was examined to address this directly. In these reactions with QueE, the percentage of D_2_O was maintained at 78 to 95% (*v/v*). As shown in Figure 8*A*, with DT and in the presence of CPH_4_, multiple deuterium atoms are incorporated into dAdo. In contrast, with YkuN/FPR/NADPH or FMN/NADPH as the reducing system, the predominant form of dAdo is unlabeled (see Fig. 8, *B* and *C*). In the absence of CPH_4_ and with DT as the reductant, the dAdo is a mixture of unlabeled and singly deuterated species (Fig. 8*A*). By contrast, with YkuN/FPR/NADPH or FMN/NADPH as reductant, the pool of enriched dAdo is almost exclusively unlabeled, with at most 17% of the dAdo pool being singly deuterated (Fig. 8, *B* and *C*). These observations suggest that multiply deuterated dAdo observed with DT under catalytic conditions results from a reductant-sensitive step that is only accessible in the presence of the substrate.

To further probe this, we carried out a control experiment in which QueE was initially reduced with DT, excess reductant was removed, and QueE was assayed as described above. If the multiply deuterated species resulted from the excess DT, one would expect that with the pre-reduced enzyme, the majority of the dAdo species will be unlabeled. As with above, in the absence of DT (see Fig. S7*B*), the pre-reduced QueE assays produced a dAdo profile resembling those observed with the milder reducing systems YkuN/FPR/NADPH and FMN/NADPH (see Fig. 7, *B and C*), with the unlabeled dAdo species present predominantly in the dAdo pool. In the presence of DT, the dAdo profile with the pre-reduced QueE is the same as observed in Figure 7*A*. In these experiments, control incubations with just the flowthrough from the final desalting step show only unlabeled dAdo (Fig. S7*C*).

### DT intervenes in the reaction by reducing the CPH_4_ radical

The data shown above clearly demonstrate that multiple deuteration of dAdo occurs only under catalytic conditions and *only with* DT as reductant. Two possible pathways by which turnover conditions would lead to multiply deuterated dAdo were discussed above (illustrated in Figs. 5 and 6). In the first (Fig. 5), the initially formed substrate radical can be intercepted by DT to generate deuterated CPH_4_. This model predicts that bulk CPH_4_ becomes enriched with deuterium under catalytic conditions with DT as a reductant.

**Figure 9:**
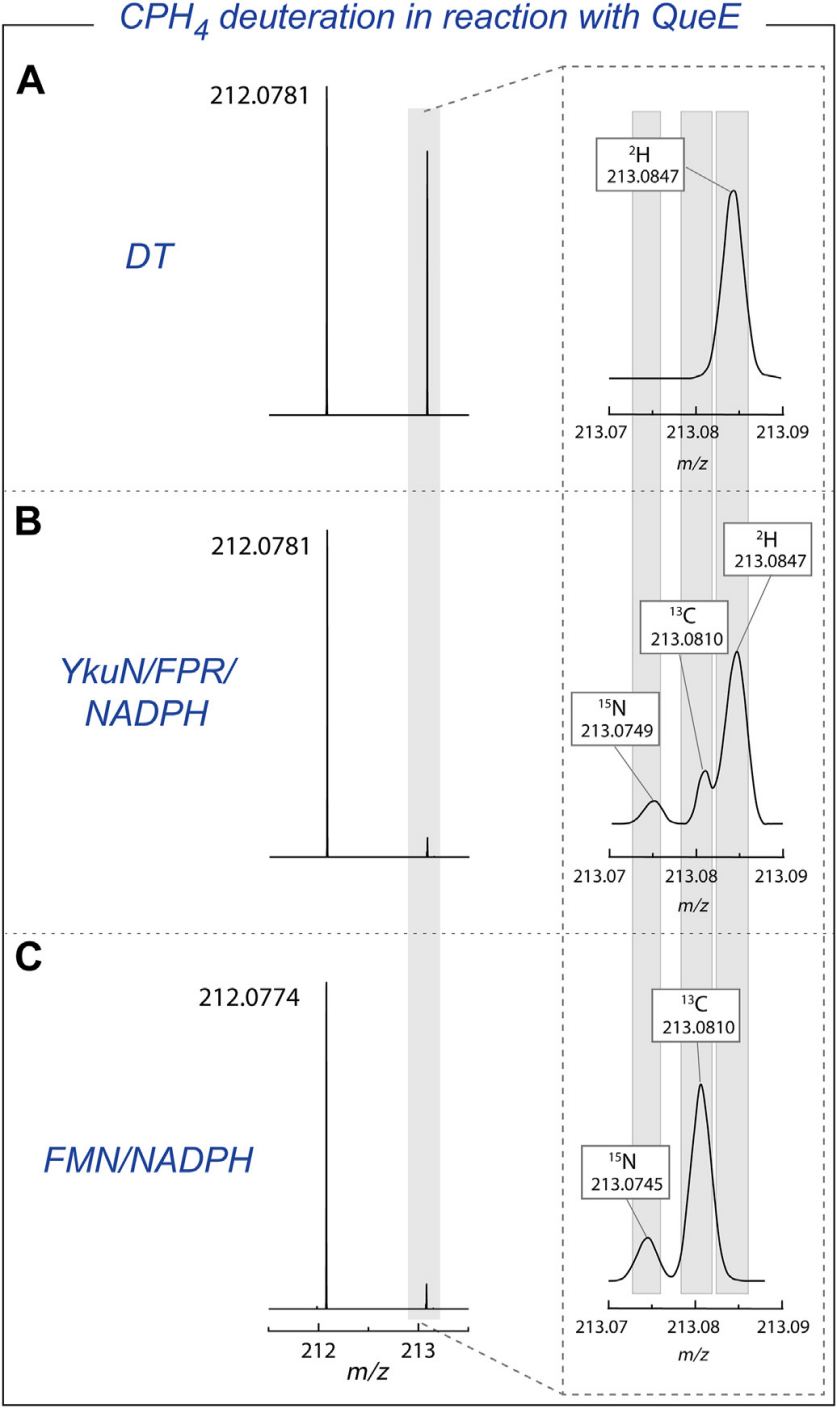
**CPH_4_ is enriched with deuterium during the reaction with DT as a reductant.** The figure shows the MS data of CPH_4_ in the reaction with QueE in the presence of (A) DT, (B) YkuN/FPR/NADPH, or (C) FMN/NADPH and was incubated for 18 h. In the panel on the *right* side, the region corresponding to the isotope peaks is expanded to show there is no deuteration into CPH_4_ when FMN/NADPH is used to activate QueE. However, a small amount of enrichment is observed with YkuN/FPR/NADPH.

To test this, we monitored the deuterium content of bulk CPH_4_ after incubation with DT, YkuN/FPR/NADPH, or FMN/NADPH to reductively activate QueE. These reactions were analyzed after 18 h. When the reaction contained QueE, SAM and CPH_4_ with DT as the reducing agent, bulk CPH_4_ was observed to incorporate deuterium (Fig. 9*A*). Deuteration of CPH_4_ was also observed in reactions with YkuN/FPR/NADPH as well (Fig. 9*B*), albeit to a much lesser extent compared to DT. As calculated using at least three replicates, ∼6% of the dAdo pool is deuterated with YkuN/FPR/NADPH, compared to ∼65% deuteration with DT. By contrast, with the FMN/NADPH reducing system, almost no multiply deuterated dAdo is observed (Fig. 9*C*). These trends mirror the appearance of multiply deuterated dAdo under catalytic conditions with the respective reducing systems and are consistent over at least three replicates. Control reactions where QueE was not present show that in the timeframe of the experiment, only a small fraction of the CPH_4_ pool is deuterated (see Figs. S8 and S9), suggesting that non-enzymatic reduction of trace amounts of oxidized CPH_4_ is not the source of deuterium enriched bulk CPH_4_ under catalytic conditions.

## Discussion

QueE catalyzes the complex radical-mediated ring contraction in CPH_4_ to form CDG, a key intermediate in the biogenesis of 7-deazapurine containing natural products. In the reaction of QueE, the dAdo· activates CPH_4_ by abstracting an H-atom from its C-6 position, which initiates the radical-mediated transformation to the product (56). The formation of dAdo· occurring through the reductive cleavage of SAM is a hallmark of nearly all rSAM superfamily members and has been suggested to include Ω, an organometallic intermediate (28, 61). The reducing equivalents required to activate rSAM enzymes for the cleavage of SAM *in vitro* are traditionally derived from inorganic reductants like DT, titanium (III) citrate, or the “biological” reducing system of flavodoxin/flavodoxin reductase/NADPH. Recent studies have posited that this reductive activation can likely occur *in vivo* by any species that can undergo one-electron oxidation (40, 62, 63).

The dAdo· that forms from the reductive cleavage of SAM can have different fates. In the abortive cleavage pathway, the dAdo· can be quenched to dAdo in a reaction that is uncoupled from the formation of the product. In the catalytic cycle, dAdo· abstracts an H-atom from the substrate. In rSAM enzymes that use SAM stoichiometrically, the H-transfer from the substrate quenches dAdo· to form dAdo (64, 65). However, in QueE and the subset of rSAM enzymes that use SAM catalytically, dAdo· is regenerated by H-transfer to generate the product and recombined with L-Met to reform SAM and reduce the cluster to its +1 state. In this manuscript, we demonstrate the existence of an alternate shunt pathway in QueE for the formation of dAdo involving quenching of the substrate intermediate.

The radical intermediates that form in the reactions catalyzed by rSAM enzymes are generally thought to be protected from capture by the active site (21, 22, 66, 67, 68). However, several previous studies have shown that shielding is not absolute. For example, Begley and colleagues have reported the ability to “trap” catalytic intermediates in MqnE by various reagents, including DT, molecular oxygen, and spin traps (69). In NosL, the shunt product 3-methylindole is produced instead of the native product 3-methylindole-2-carboxylic acid in the presence of DT (70). Studies of the rSAM enzyme Dph2 revealed that a DT-quenched radical product forms an organometallic complex with the RS cluster, highlighting a direct interference of DT in these systems (71). Outside of the rSAM superfamily, DT has been reported to interfere in the study of nitrogenases, where the DT-derived SO_2_^·^^−^ radical is involved in the slow reduction of the iron center (72, 73, 74). A recent report also highlights how DT could impede the electrochemical characterization of nitrogenases (75). The electrochemical activity of SO_3_^2−^, a decomposition product of DT, is indistinguishable from that of the reaction mixture containing nitrogenase enzyme in the presence of DT, confounding the analysis of cyclic voltammetry analysis of the reaction mixtures. These examples underscore the potential for DT to interfere directly with the reactions catalyzed by metalloenzymes.

Recently, we reported that a mixture of FMN/NADPH can reductively activate QueE and PapB, two unrelated rSAM enzymes that catalyze distinct transformations (40). PapB is involved in forming one or more thioether crosslinks in the ribosomally produced and posttranslationally modified polypeptide, PapA, which is vastly different from the transformation carried out by QueE. Nevertheless, the FMN/NADPH reducing system has also been shown by Allen and colleagues to effectively activate methylthiotransferases from a thermophilic *Ca.* Methanophagales anaerobic methanotroph (C4B56_06395), and from a hyperthermophilic methanogen *Methanocaldococcus jannaschii* (MJ0867) (41). In the experiments with QueE described in this paper, we observe that, unlike DT, the use of FMN/NADPH to reductively activate the enzyme does not lead to the formation of multiply deuterated dAdo. Similar observations were made with the biological reducing system of YkuN/FPR/NADPH. The deuteration pattern of dAdo is likely because FMN/NADPH and YkuN/FPR/NADPH cannot intercept reactive catalytic intermediates. We posit that at least some of the reports documenting the inability to observe turnover with DT as a reductant may be traced to the off-pathway shunt products formed by reaction with DT (32, 34).

Herein, we demonstrate that in QueE, uncoupled production of dAdo is not limited to only abortive cleavage of SAM in binary complexes with the enzyme but can also occur in ternary complexes that include the substrate undergoing turnover. The CPH_4_-based substrate radical appears to be prone to capture by reduction by DT, leading to the formation of the starting reactant CPH_4_. However, in D_2_O, the formation of the CPH_4_ also leads to the deuteration of the substrate, which in subsequent turnovers leads to the capture of the deuterium into dAdo. Since SAM is used catalytically in QueE, dAdo resulting from the reaction is multiply labeled.

The results reported here with QueE suggest that when studying rSAM enzymes, one must carefully consider the choice of reductant (76). A non-physiological reductant can establish shunt reactions in the radical-mediated transformations catalyzed by rSAM enzymes. In fact, a lack of activity in systems when DT has served as a reductant suggests that the use of this reagent could be detrimental to activity and can be replaced by convenient alternatives, such as FMN/NADPH.

## Experimental procedures

### Preparation of QueE, GCH I, QueD, and CPH_4_, SAM, YkuN, FPR

The overexpression and purification of the proteins QueE, GCH I, QueD, YkuN, and FPR were carried out as described previously (77). SAM (78) and CPH_4_ (79) were synthesized enzymatically as described previously. These were lyophilized and dissolved in a minimal amount of H_2_O to make the stock solutions used in the reaction. Commercial SAM was used in the assays containing D_2_O to maximize the D_2_O content in the reaction.

### QueE activity assays in H_2_O

The reactions with QueE were carried out in the dark in an anaerobic Coy chamber, and the progress of the reactions was monitored by UHPLC-MS. The assays carried out in H_2_O contained 50 mM potassium phosphate (KPi) buffer pH 7.4, 2 mM MgSO_4_, 25 μM QueE, 2 mM CPH_4,_ and 2 mM SAM, and were incubated at room temperature for 18 h. The reducing agent used for the QueE reactions was either 2 mM DT, 25 μM YkuN/25 μM FPR/2 mM NADPH, or 50 μM FMN/2 mM NADPH. These reactions were incubated at room temperature for 18 h and quenched by the addition of 3% TCA. The resulting precipitate was centrifuged at 21,000*g* for 10 min before analyzing the supernatant by LC-MS.

### QueE activity assays in D_2_O

For the reactions carried out in D_2_O, we tried to maximize the D_2_O content used in the assays to 78 to 95% D_2_O (*v/v*). The KPi buffer stock (1 M) was prepared in D_2_O aerobically by adding potassium phosphate monobasic, and sodium deuteroxide (Cambridge isotope labs, D, 99.9%, 40% (*w/v*) in D_2_O) was added dropwise to adjust the pH to 7.0 (pD 7.4) (80). This buffer was then flash-frozen in liquid N_2_ and lyophilized. The resulting powder was brought into the anaerobic chamber and dissolved in the same volume of anaerobic D_2_O. QueE, CPH_4_, and FPR were not exchanged into a deuterated buffer due to the small volumes used in the reaction. MgSO_4_ was prepared by repeatedly dissolving commercial anhydrous MgSO_4_ in D_2_O, flash-freezing it in liquid N_2_, and lyophilization. This process was carried out three times, yielding a white powder, which was brought up to volume with D_2_O inside the anaerobic chamber. The stock solutions of DT, SAM, FMN, and NADPH were prepared in D_2_O under anaerobic conditions and used in the reactions. Purified YkuN was buffer exchanged using 10 ml Econo-Pac 10DG desalting Column (Bio-Rad). The desalting column was equilibrated with 50 mM KPi pD 7.4 and 2 mM DTT buffer, and the concentrated protein was loaded onto the column. This protein was eluted using the equilibration buffer. The yellow fractions were pooled and concentrated using 10 kDa MWCO centrifugal filters to a total volume of 500 μl. The protein was then aliquoted, flash-frozen in liquid N_2_, and stored at −80 °C until further use. For the QueE reactions, the assay mixture contained 50 mM KPi buffer (pD 7.4), 2 mM MgSO_4_, 25 μM QueE, 2 mM CPH_4,_ and 2 mM SAM. The reducing agents used for the reactions were either 2 mM DT, 25 μM YkuN/25 μM FPR/2 mM NADPH, or 50 μM FMN/2 mM NADPH. These reactions were incubated at room temperature for 18 h and quenched by the addition of 3% TCA. The resulting precipitate was centrifuged at 21,000*g* for 10 min before analyzing the supernatant by LC-MS.

### Pre-reduction of QueE for assays in D_2_O

Purified QueE was incubated with 10 mM DT for 15 min. The protein solution was desalted using a manually packed 50 ml Bio-Gel P-6 DG Gel (Bio-Rad #1500738) and exchanged into a buffer containing 50 mM PIPES-NaOH (pH 7.4) and 2 mM DTT. An aliquot of this protein (∼100 μl) was added to a 10 kDa MWCO centrifugal filter and diluted by the addition of ∼400 μl buffer containing 50 mM PIPES-NaOH (pH 7.4) and 2 mM DTT to a total volume of ∼500 μl. This mixture was concentrated by centrifuging at 5000*g* in short 5-min bursts while repeatedly mixing the contents with a micropipette until the final volume was ∼100 μl. The resulting concentrated protein was diluted and concentrated as above three more times to remove traces of DT. The flowthrough from the final centrifugation step was used as a control in the assays shown in Figure S7*C*, and the resultant concentrated protein was assayed and analyzed as described above.

### Analysis of QueE reactions using LC-MS

The reactions were analyzed on a Thermo Fisher Scientific Vanquish UHPLC in-line with a UV detector and a QExactive high resolution mass spectrometer. A reversed-phase C18 column was used to separate the components of the reaction. The mobile phase consisted of 0.1% trifluoroacetic acid (LC-MS grade) in water (buffer A) and 0.1% trifluoroacetic acid in acetonitrile (buffer B). The separation of the reaction components was achieved by 3 min of 100% buffer A, followed by a linear gradient to 10% buffer B over 35 min to elute the reaction components. Following the gradient elution, the column was washed for 4 min with 100% buffer B and re-equilibrated by washing in buffer A for 5 min. The flow rate was maintained at 0.2 ml/min in all runs.

For the MS2 experiments, an LC-MS method was set up to perform in-run targeted single-ion monitoring (t-SIM) with data-dependent MS2 acquisition (dd-MS2) in positive mode. This method identifies and isolates species meeting an ion intensity threshold of 5E + 4 ions, and the 6.0 *m/z* window from 251.1217 to 257.1217 *m/z* encompasses all the ions of interest. The ions with the desired mass (either dAdo or doubly deuterated dAdo) are further identified and isolated before fragmentation utilizing HCD (higher-energy collisional dissociation) with a normalized collision energy (NCE) of 35%. For these experiments, the LC separation method was modified so that dAdo was well separated from other species present in the reaction. The flow rate was maintained at 0.2 ml/min throughout. The elution gradient was set up with the same buffer A and B as described above, with multiple short steps as follows: 3 min wash at 0% B, step to 0.2% B over 0.25 min, step to 0.8% B over 0.25 min, step to 3.2% B over 0.25 min, step to 5% B over 0.25 min, step to 25% B over 3 min, step to 50% B over 3, and step to 75% B over 2 min. Following this, the concentration of B increased to 100% over 0.1 min, and the column was washed with 100% B for 3 min, followed by washing for 3 min in 0% B to re-equilibrate the column.

### Analysis of deuterium content of dAdo and CPH_4_ formed in the reaction

The theoretical *m/z* of unlabeled dAdo is [M+H]^+^ = 252.1091, mono-deuterated dAdo is [M+H]^+^ = 253.1154, doubly deuterated dAdo is [M+H]^+^ = 254.1217, and triply deuterated dAdo is [M+H]^+^ = 255.1279. Extracted ion chromatograms (EIC) traces were generated from the total ion chromatogram (TIC) using the following *m/z* ranges: unlabeled dAdo, 252.1010 to 252.1200; singly labeled dAdo, 253.1020 to 253.1280; doubly labeled dAdo, 254.1080 to 254.1280; triply labeled dAdo, 255.1140 to 255.1340. The area under these peaks for the deuterated species includes not only the area of the monoisotopic peak but also a contribution from the natural abundance isotopes of the lighter species. For example, the 253.1020 to 253.1280 encompasses all the natural abundance isotopes from the unlabeled dAdo (C_10_H_13_N_5_O_3_) as well as deuterated dAdo. To calculate the contribution from the ^2^H isotope incorporated into dAdo in the reaction in D_2_O, the theoretical isotopic natural abundance of ^13^C, ^15^N, ^2^H, and ^18^O isotopes were calculated (using https://www.envipat.eawag.ch/index.php) and their contribution subtracted (81). Data from the reactions carried out with at least three replicates were used to obtain the results shown in the figures. The average areas of each control reaction were normalized by the average area of the full reaction, and the obtained area was plotted as a bar graph. The standard deviation of the reaction replicate data was used to plot the error bars. The procedure for extracting the deuteration levels of CPH_4_ was similar, except that *m/z* ranges of 212.0700 to 212.0900 and 213.0785 to 213.0900 were used to generate the EIC for the unlabeled and singly deuterated species.

## Data availability

All data used in this study are present in this manuscript and supporting information.

## Supporting information

This article contains supporting information.

## Conflict of interest

The authors declare that they have no conflicts of interest with the contents of this article.

## Supporting information

Figures S1−S9
