# Supplementary material for: Not all 5′-deoxyadenosines are created equal: Tracing the provenance of 5′-deoxyadenosine formed by the radical S-adenosyl-L-methionine enzyme 7-carboxy-7-deazaguanine synthase
Source: J Biol Chem. 2025 Feb 25;301(4):108347. doi: 10.1016/j.jbc.2025.108347 (PMC11994313; doi:10.1016/j.jbc.2025.108347)
Supplement: Figures S1−S9 [file mmc1.docx]

**Supporting information**


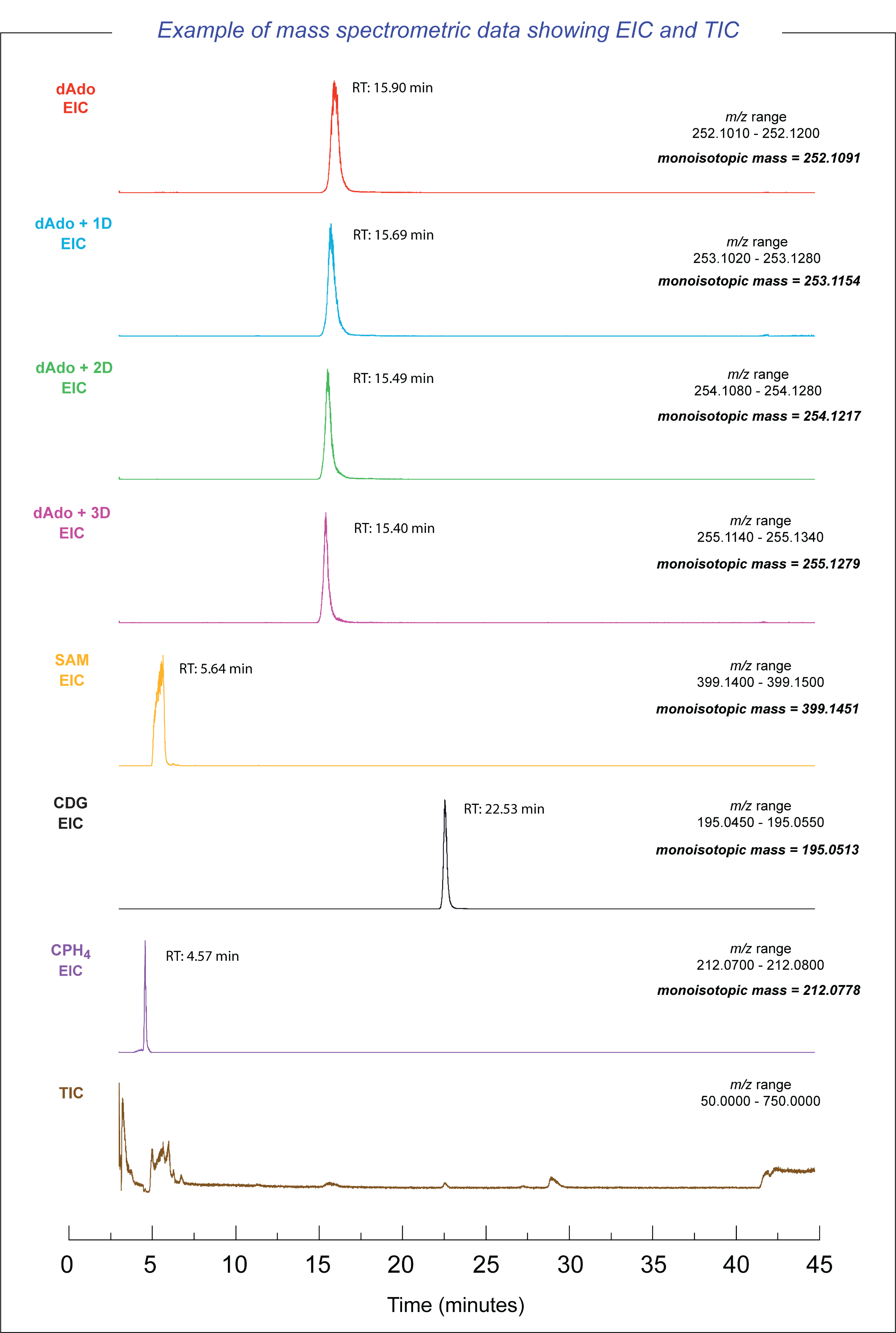


**Figure S1:** Representative LC-MS analysis of DT-activated QueE reactions. The reaction mixture contains 50 mM KPi buffer pD 7.4, 2 mM MgSO_4_, 2 mM CPH_4_, 25 µM QueE, and 2 mM SAM in 95% D_2_O. The total ion chromatograms (TIC) are shown in the bottom panel, with the extracted ion chromatograms (EIC) corresponding to the dAdo species, SAM, CDG, and CPH_4_ shown in the panels above. The theoretical *m/z* values are shown as appropriate.


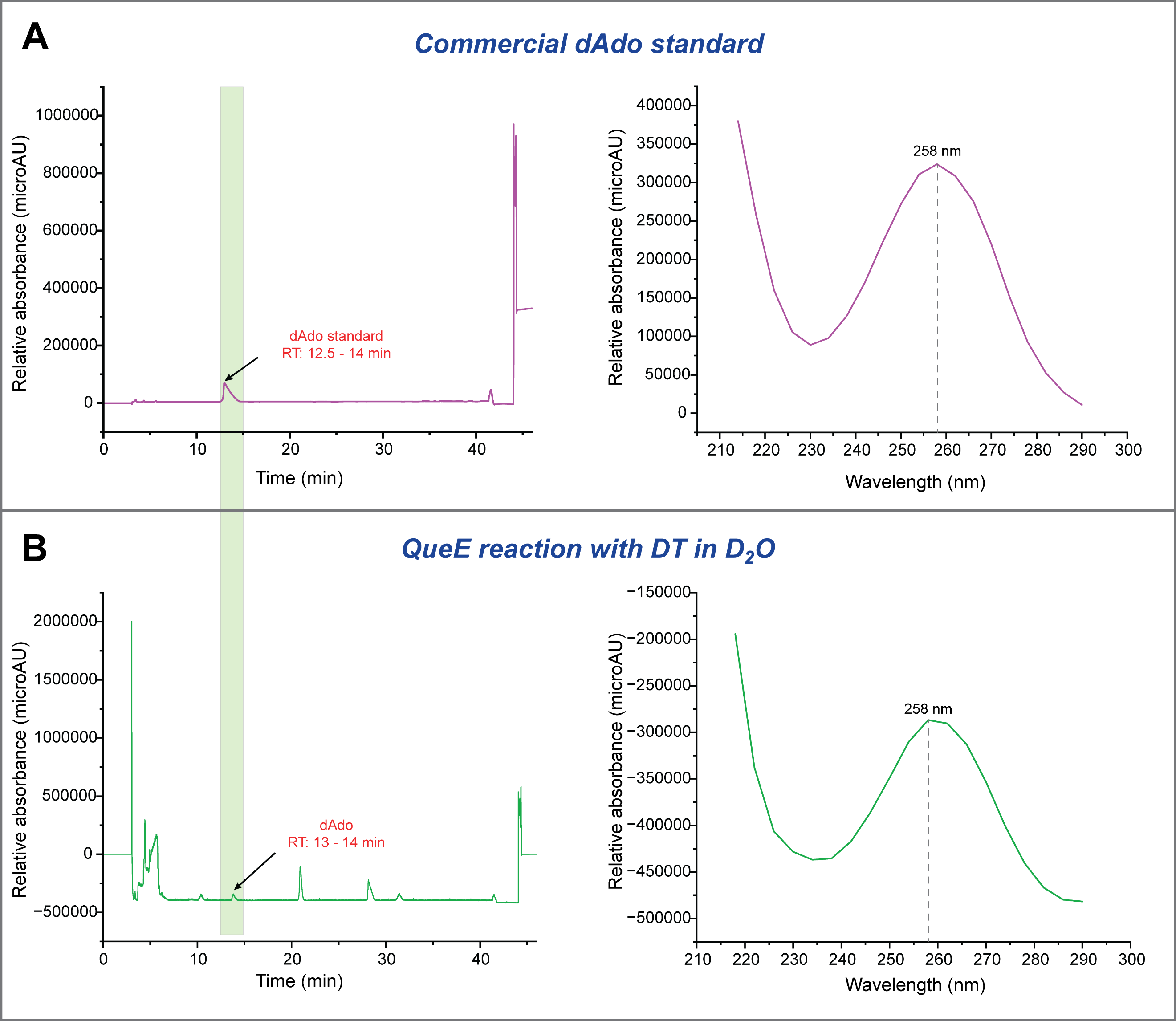


**Figure S2:** HPLC elution profiles for dAdo. (**A**) The dAdo standard control elutes 12.5-14 min and exhibits a peak at 258 nm. (**B**) The retention time and UV/visible features for dAdo formed in the QueE reaction are similar to the commercial standard. Note that the retention time for dAdo is somewhat variable from day to day (generally 13-16 min), but within any set of experiments that are analyzed as part of a group remains consistent.


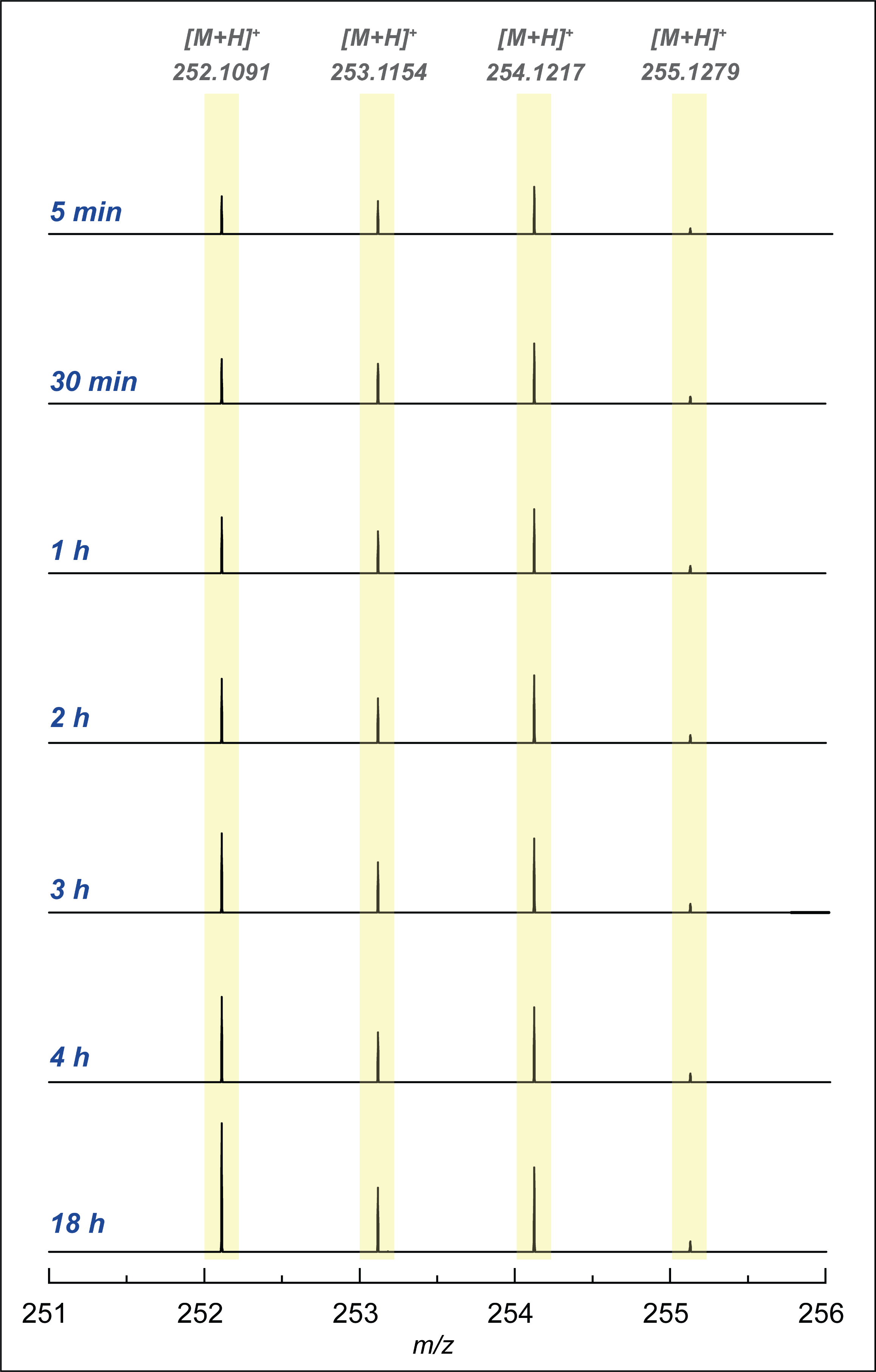


**Figure S3**: The appearance of multiply deuterated dAdo is consistent across the time course of the reaction and is even observed in the early time points of the reaction. The theoretical *m/z* are depicted in the figure, and the observed mass is within 3 ppm error with respect to the theoretical mass.


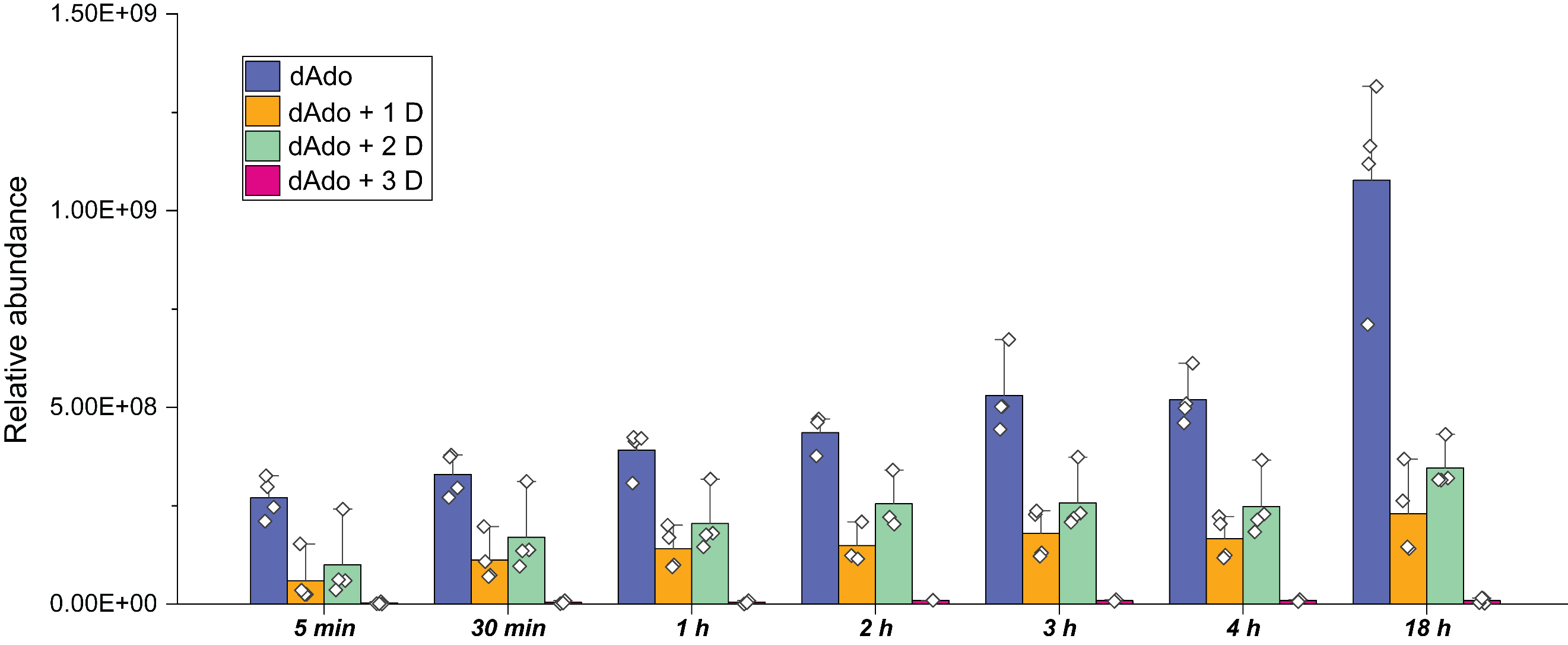


**Figure S4**: The dAdo formed in the reaction increases over time and the formation of multiply deuterated dAdo is observed from the 5 min time point. The bars correspond to the average area of each dAdo species obtained at the respective time point, as obtained from the EIC (see *Experimental procedure*). In each set of experiments, the bar graphs represent the area under the EIC of the corresponding dAdo species. The standard deviation in the data is depicted in the error bars.


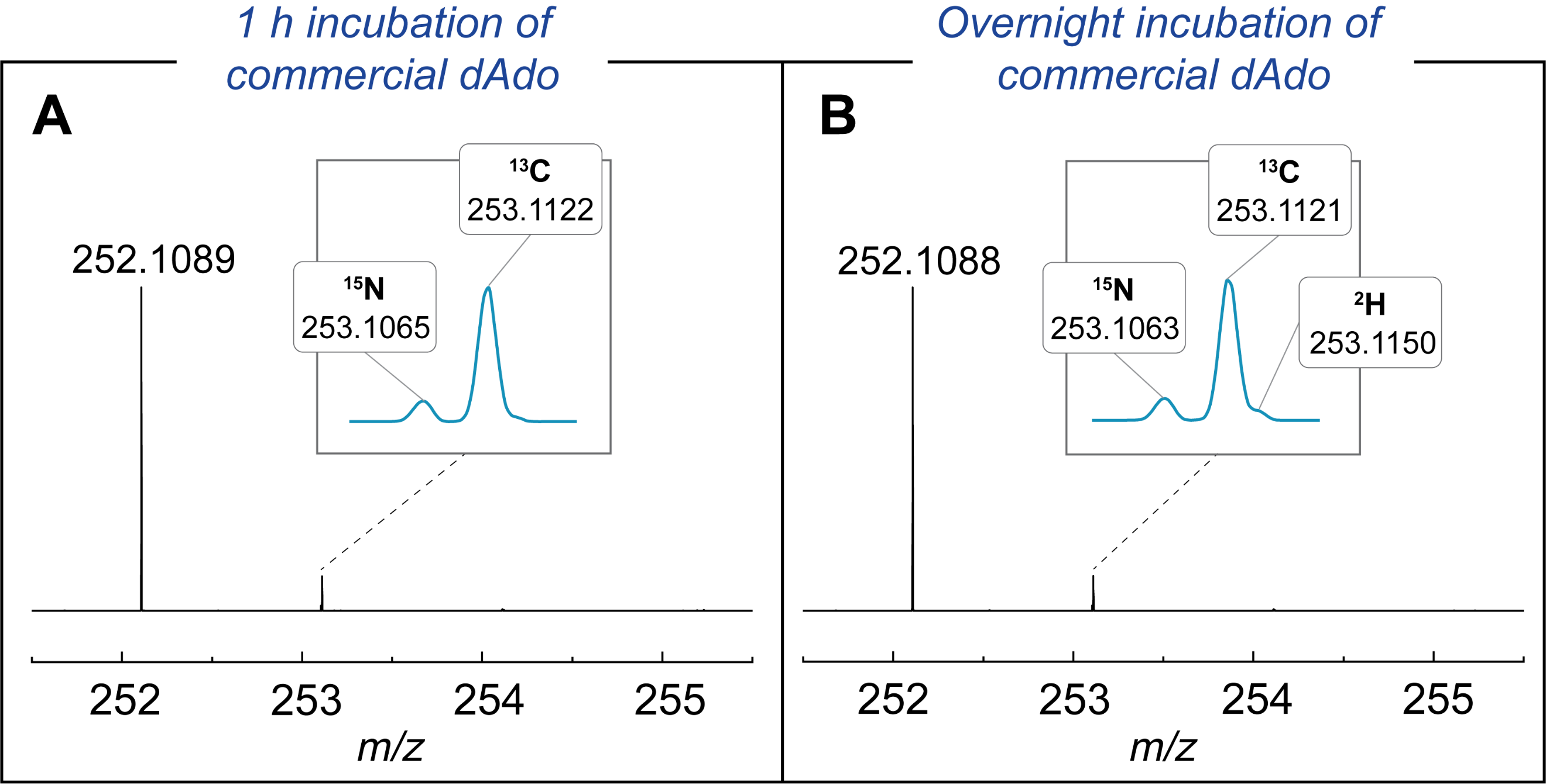


**Figure S5**: The incorporation of deuterium labels on commercial dAdo after (**A**) 1 h and (**B**) overnight incubation is not sufficient to account for the multiply deuterated dAdo observed in **Figure 3** (**C**). The reaction mixture contained 2 mM commercial dAdo in the presence of 50 mM KPi pD 7.4, 2 mM L-Met, 2 mM MgSO_4_, 2 mM CPH_4_, 25 µM QueE and 2 mM DT in 95% D_2_O.


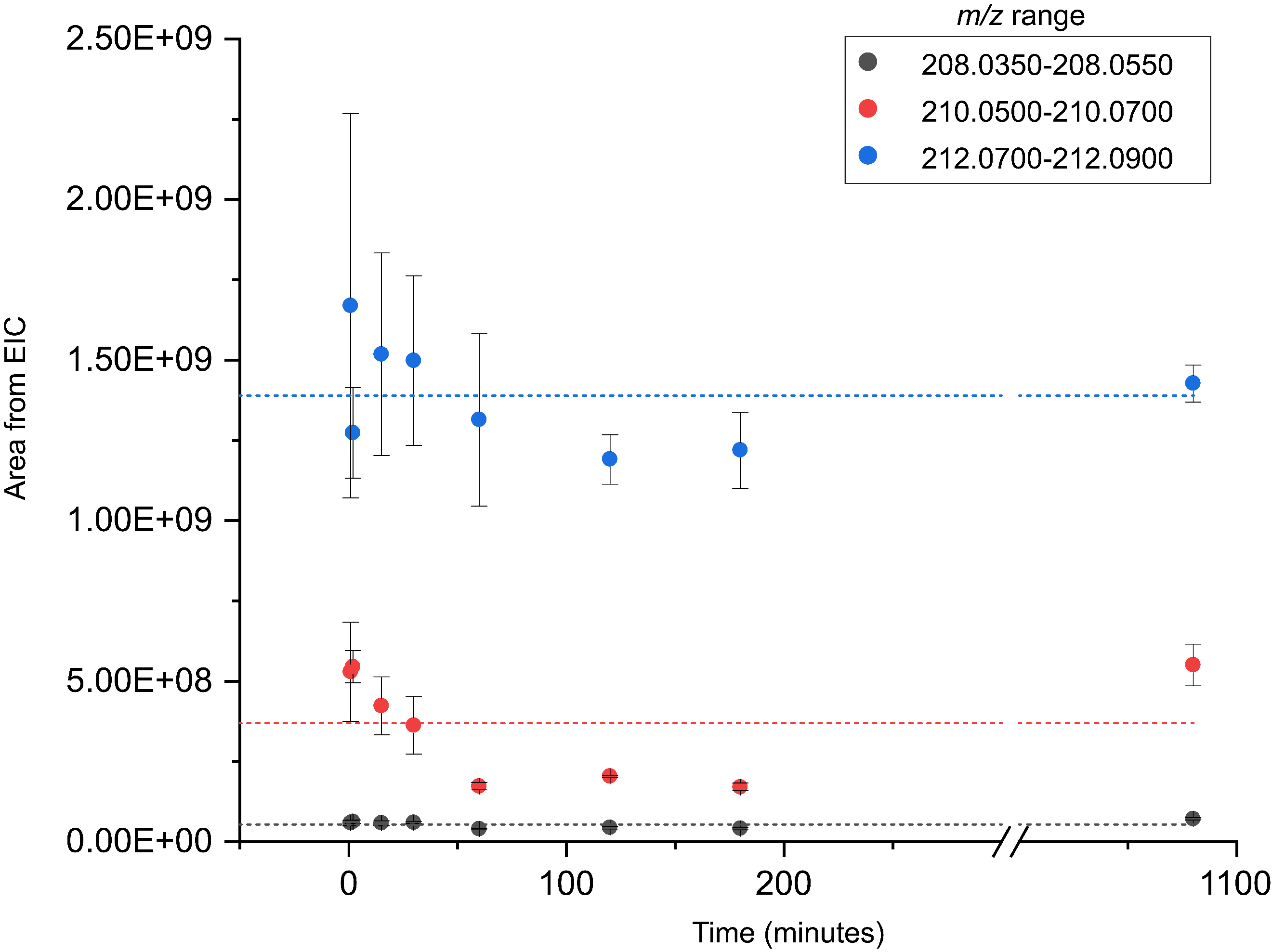


**Fig S6**: Monitoring CPH_4_ and oxidized analogs. An aliquot of a CPH_4_ stock containing approximately 30% oxidized species was tested for reduction by incubating it with DT under the assay conditions in the absence of QueE. These assays contained 50 mM KPi buffer (pD 7.4), 2 mM MgSO_4_, 2 mM CPH_4_ and 2 mM SAM in D_2_O in the presence of 2 mM DT. Time points were taken and analyzed by LC-MS as described for CPH_4_. The area under the EIC of fully oxidized CPH_4_ (theoretical *m/z* = 208.0465), semi-reduced CPH_4_ (theoretical *m/z* = 210.0621), and CPH_4_ (theoretical *m/z* = 212.0778) were calculated and plotted to see the time evolution of the different species. The error bars represent standard deviation in the data and the dotted reference lines correspond to the average area of the EIC of all the sampled time points.


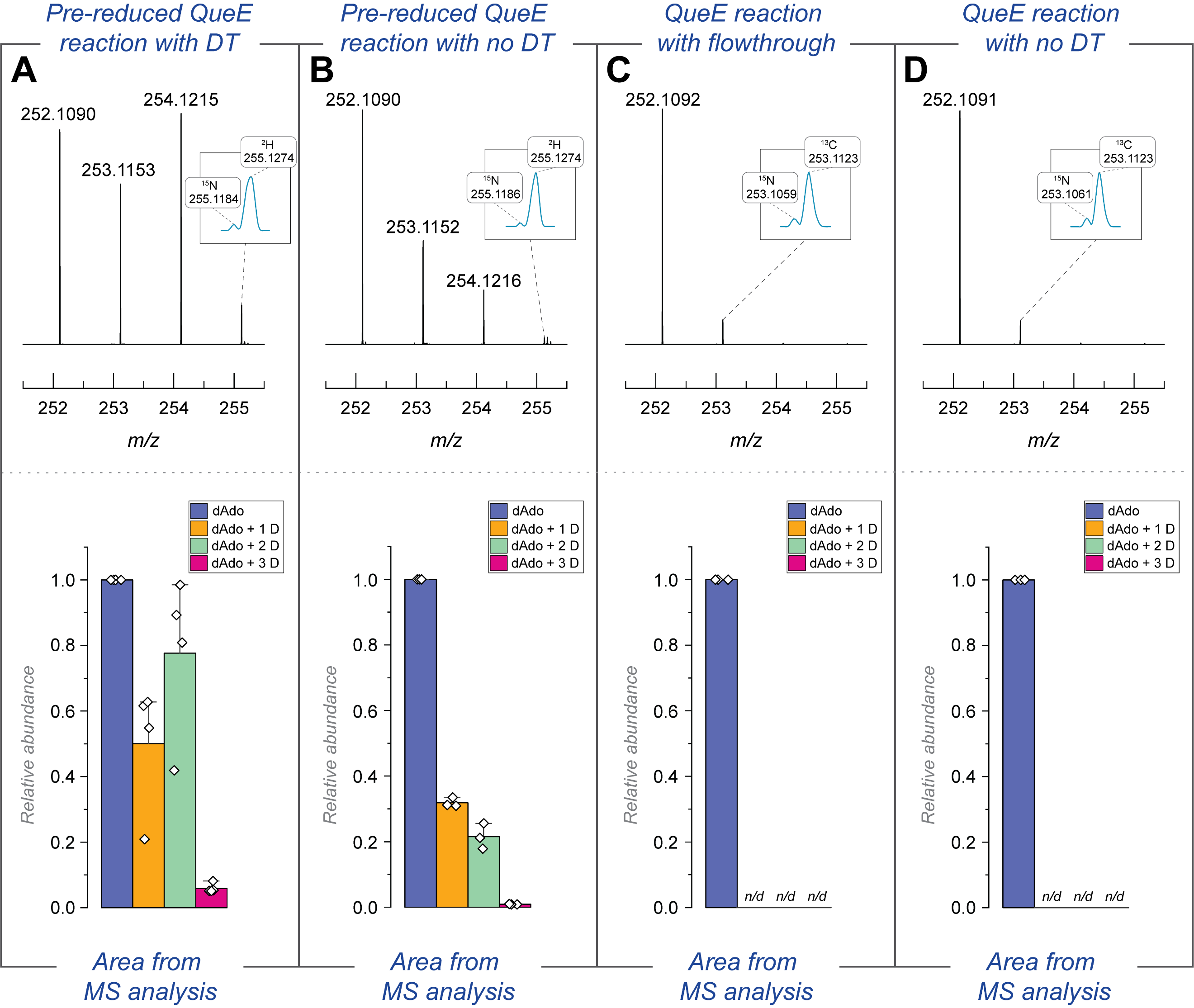


**Figure S7**: Comparison of deuterium content of dAdo in the presence or absence of DT with pre-reduced QueE. QueE was pre-reduced with DT and the excess reductant was removed as described in *Methods*, before being used in these assays. (**A**) The reaction of pre-reduced QueE with CPH_4_, SAM, and DT showed the previously observed multiply deuterated dAdo (see **Fig. 7A**). (**B**) When pre-reduced QueE was incubated in reactions containing CPH_4_ and SAM, the resulting dAdo was largely unlabeled or singly deuterated. Some doubly deuterated species were also observed. Control assays in the presence (**C**) of a small aliquot of the flow through from the final desalting step was added into the reaction, or its absence (**D**), show no deuterated dAdo species. In each set of experiments, the area under the EIC of the deuterated species of dAdo was normalized to the area of unlabeled dAdo species and the error bars represent standard deviation of the data.


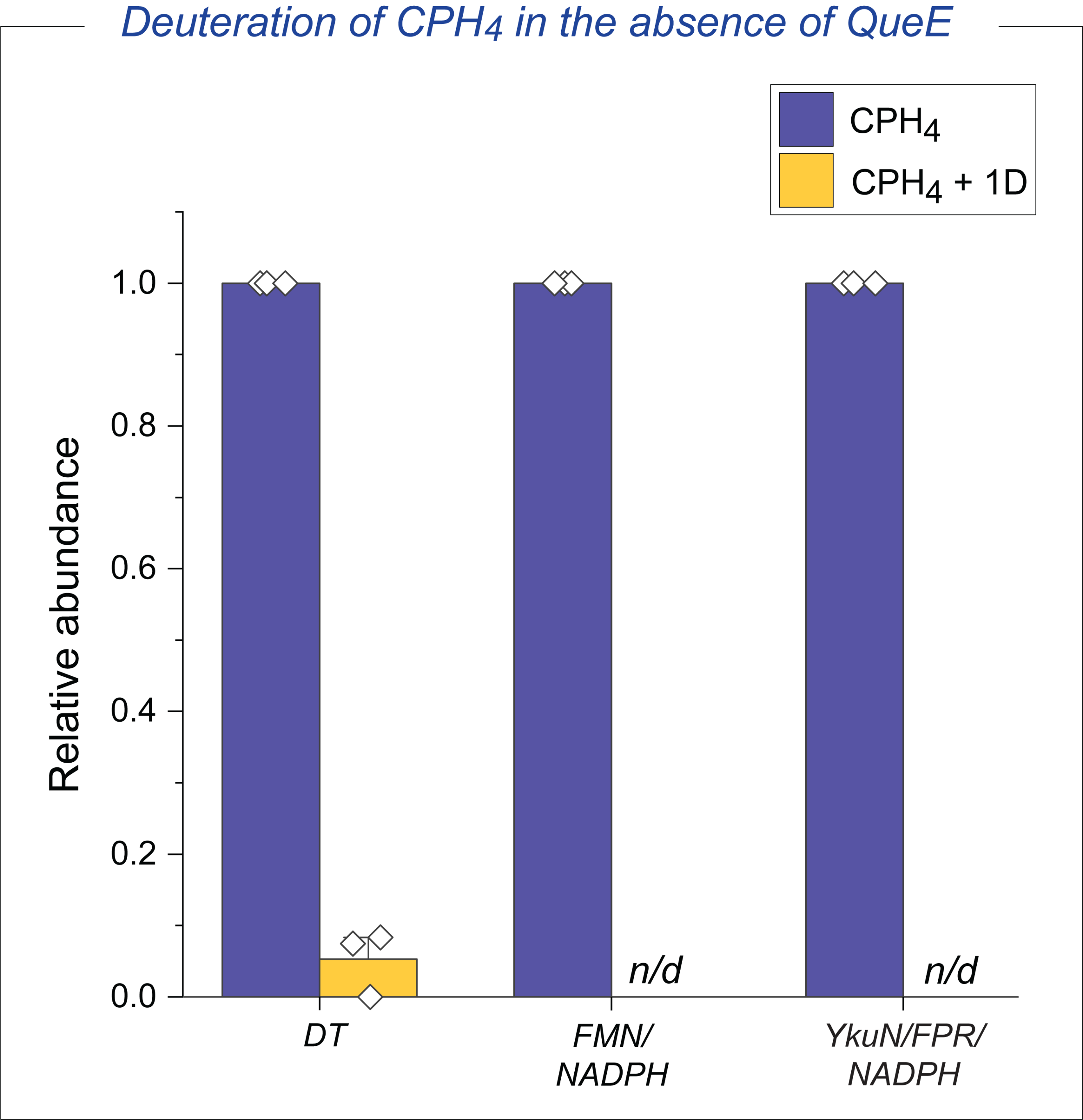


**Figure S8**: CPH_4_ is not deuterated if incubated under reaction conditions in the absence of QueE for 18 h. Peak areas in the EIC for CPH_4_ and deuterated CPH_4_ were analyzed as described in the *Methods* section. A small amount (<10%) of the stock appeared to be deuterated when DT was used as the reducing agent. No evidence for deuteration of CPH_4_ is present with FMN/NADPH or YkuN/FPR/NADPH. In each set of experiments, the area under the EIC of the deuterated species of CPH_4_ was normalized to the area of unlabeled CPH_4_ species and the error bars represent standard deviation of the data.


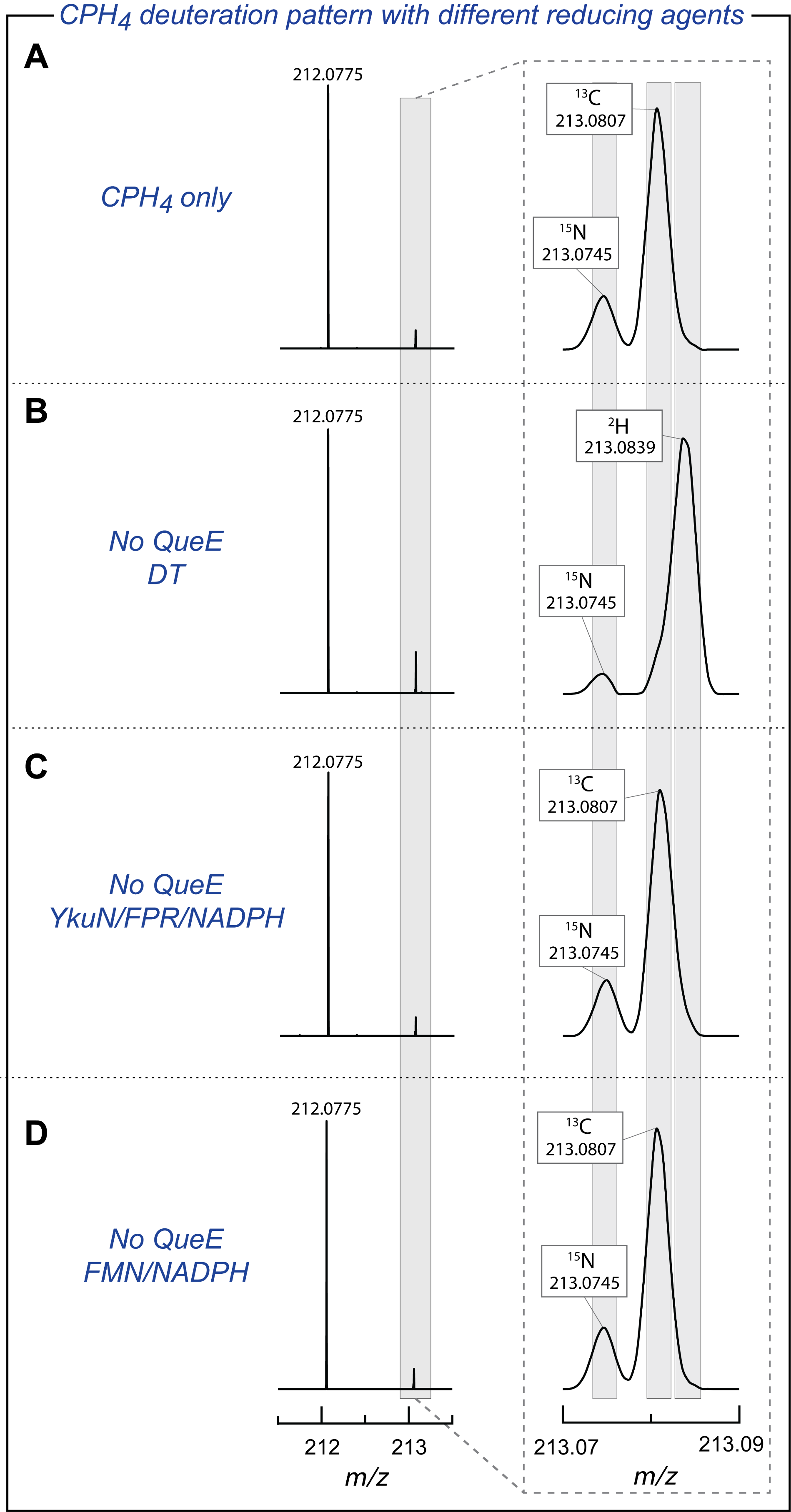


**Figure S9:** The MS traces compare the deuteration of CPH_4_ in the absence of QueE with different reducing agents present in the reaction carried out in D_2_O. No deuteration of CPH_4_ is observed with YkuN/FPR/NADPH or FMN/NADPH; however, a small amount of CPH_4_ is deuterated with DT. This is likely due to the reduction of oxidized CPH_4_ by DT, which then incorporates deuterium labels.
